# Gentamicin–Ascorbic Acid Encapsulated in Chitosan Nanoparticles Improved In Vitro Antimicrobial Activity and Minimized Cytotoxicity

**DOI:** 10.3390/antibiotics11111530

**Published:** 2022-11-02

**Authors:** Mohamed A. Abdel-Hakeem, Ahmed I. Abdel Maksoud, Mohammed Abdullah Aladhadh, Khalid Abdulrahman Almuryif, Rafaat M. Elsanhoty, Dalia Elebeedy

**Affiliations:** 1 Department of Pharmaceutical Biotechnology, College of Biotechnology, MISR University for Science and Technology, Giza 3236101, Egypt; 2 Department of Industrial Biotechnology, Genetic Engineering and Biotechnology Research Institute (GEBRI), University of Sadat City, Sadat City 32897, Egypt; 3 Department of Food Science and Human Nutrition, College of Agriculture and Veterinary Medicine, Qassim University, Buraydah 51452, Saudi Arabia; 4 Ministry of Health, Kingdom of Saudi Arabia, Buraydah 51452, Saudi Arabia

**Keywords:** nano-drug delivery, antimicrobial drugs, gentamicin sulfate (GM), chitosan nanoparticles (CSNPs), FTIR

## Abstract

Nano-drug delivery is a promising tactic to enhance the activity and minimize the cytotoxicity of antimicrobial drugs. In the current study, chitosan nanoparticles (CSNPs) were used as a carrier for the delivery of gentamicin sulfate (GM) and ascorbic acid (AA). The particles were synthesized by ionotropic gelation method and characterized by FT-IR, Zeta potential, and transmission electron microscope imaging. The obtained particles were evaluated for their in vitro antimicrobial activity and cytotoxicity. The prepared particles (GM–AA–CSNPs) under the optimal condition of 4:1:1 of chitosan to drug ratio showed encapsulation efficiency and loading capacities of 89% and 22%, respectively. Regarding biological activities, GM–AA–CSNPs showed a lower minimum inhibitory concentration (MIC) than free gentamicin sulfate and GMCSNPs mixture without presenting cytotoxicity against normal cells (HSF). Moreover, the GM–AA–CSNPs did not exhibit hemolytic activity. These results highlight that the GM–AA–CSNPs are confirmed as a hopeful formula for future investigations on the development of antimicrobial preparations.

## 1. Introduction

Maintaining human health is one of the sustainable development goals that researchers seek to achieve. In this context, searching for new drugs or improving the efficiency of known drugs is conducted. Drug delivery is one of the most critical issues in the drug industry and faces many challenges, such as toxicity, low bioavailability, short half-life, and low potential to reach the target site [1]. Unfortunately, many drugs in the clinical phase fail to achieve promising clinical results due to failing in one of these challenges [2].

Recently, nanotechnology has emerged as a promising approach to drug delivery [3]. Indeed, there has been unexpected growth of published work dealing with nano-drug delivery of antimicrobial agents, antitumor drugs [4], and others [5].

Despite their small size, nanoparticles provide a substantial functional surface, making them ideal for adsorption, binding, and transporting molecules such as proteins, drugs, and natural products. Additionally, nanoparticles efficiently overcome biological barriers, enhancing drug bioavailability and permanence time at the target tissue, preventing drug degradation, and achieving a sustained release pattern [6]. 

The aminoglycoside antibiotic gentamicin (GM) is used to treat various bacterial infections in the human body [7]. Nevertheless, GM has fast clearance and low bioavailability, so it is conventionally administrated via multiple dosing, causing severe side effects such as ototoxicity and nephrotoxicity [8].

Ascorbic acid (AA) is an essential water-soluble vitamin related to many health benefits [9]. Besides its function as a cofactor with many enzymes, AA is an antioxidant that reduces reactive oxygen species (ROS) into stable molecules. AA was investigated as a protective additive to reduce gentamicin toxicity [10]. In this regard, the loading of a gentamicin–ascorbic acid combination in nanocarriers could be interesting in reducing adverse effects and enhancing the treatment of bacterial infections.

Several studies investigated the formulation of gentamicin-loaded nanoparticles based on PLGA [11,12], CaCO_3_ [13], Polylactide-co-glycolide-co-Polyethylenglycol (PLGA-PEG) blends [14], and chitosan [15]. However, to our knowledge, no study has investigated the combined antimicrobial activity and cytotoxicity of GA and AA. The current work has investigated the preparation, antimicrobial activity, and cytotoxicity of gentamicin–ascorbic acid encapsulated in chitosan nanoparticles.

## 2. Results

### 2.1. Loading Capacity and Encapsulation Efficiency

**Figure 1:**
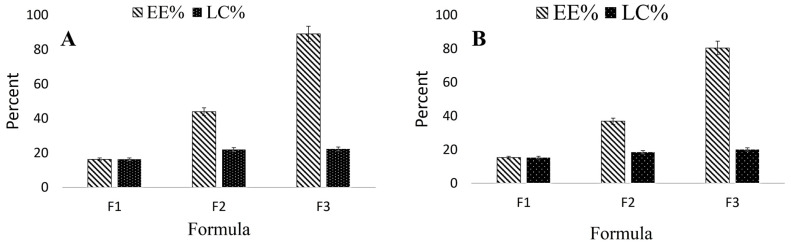
EE% and LC% of GM (**A**) and AA (**B**) in GM–AA–CSNPs. CS:GM:AA ratio F1 (1:1:1)–F2 (2:1:1)–F3 (4:1:1).

In this study, CS was mixed with different GM and AA ratios (1:1, 2:1, and 4:1). The results revealed that the EE% and LC% increased as the polymer ratio increased (Figure 1). In detail, Figure 1 demonstrates that the EE% values of GM and AA were 16.25 ± 1.9% and 15.24 ± 2.4% at a CS: GA: AA ratio of 1:1:1, respectively. When the ratio of CS increased to 4 the EE% was raised to 89.05 ± 4.70 and 80.42 ± 3.44 for GM and AA, respectively. Moreover, the LC% of nanoparticles achieved the highest value at the same CS: GA: AA ratio.

### 2.2. In Vitro Release Study

**Figure 2:**
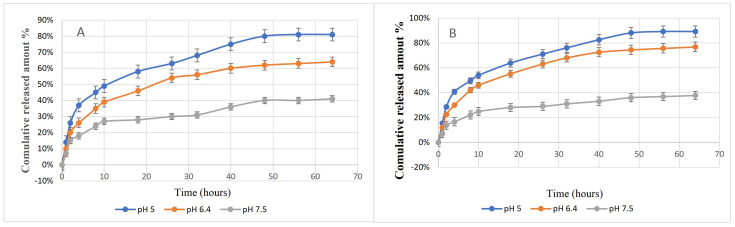
In vitro release profile of GM (**A**) and AA (**B**) from CSNPs at different pH values.

Figure 2 illustrates the in vitro release profiles of GM and AA. At the three pH values, the release profiles had two major phases. Initially, there was a rapid release in the first ten hours, then the drugs were slowly released with time until achieving the highest amount after approximately 60 h. Moreover, the highest release percentages (~82% and 89%) were detected at pH 5 for GM and AA, respectively. 

### 2.3. Characterization of GM–AA–CSNPs

**Figure 3:**
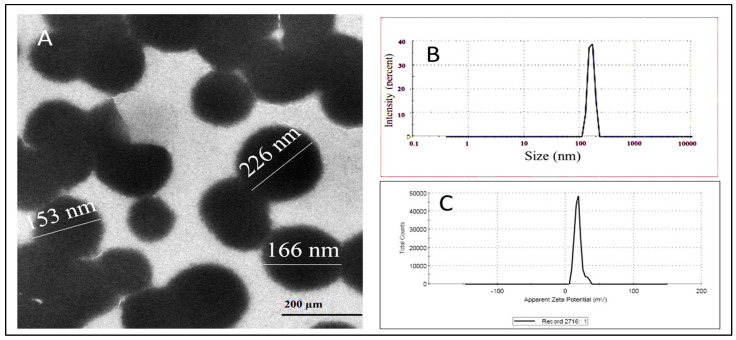
Characterization of GM–AA–CSNPs. (**A**): TEM image; (**B**,**C**): Zeta potential.

**Figure 4:**
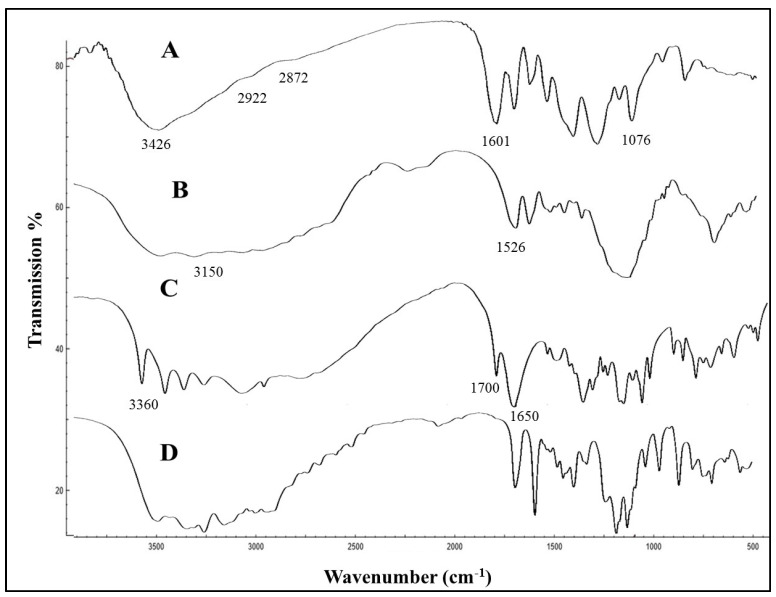
FTIR spectra of CS (**A**) showed 3426 cm^−1^ (–OH and –NH_2_), 2922 cm^−1^, 2872 cm^−1^ (–CH), 1601 cm^−1^ (–NH_2_), and 1076 cm^−1^ (C–O–C). GM (**B**) showed characteristic peaks at 3150 cm^−1^ (–OH and –NH_2_ stretching) and 1526 cm^−1^ (C=N). AA (**C**) showed characteristic peaks at 3360 cm^−1^ (–COOH), 1700 cm^−1^, and 1650 cm^−1^ (C=O). GM–AA–CSNPs (**D**).

The TEM image (Figure 3A) shows that the GM–AA–CSNPs were nearly spherical, and the average size was about 150–250 nm. Moreover, GM–AA–CSNPs showed a zeta potential of +30.01 mV and an average hydrodynamic size of 278 nm (Figure 3B and Figure 3C, respectively). Finally, the FTIR spectra of CS, GM, AA, and GM–AA–CSNPs showed the basic structural features of CS (Figure 4A) at 3426 cm^−1^ (–OH and –NH_2_), 2922 cm^−1^, 2872 cm^−1^ (–CH), 1601 cm^−1^ (–NH_2_), and 1076 cm^−1^ (C–O–C). GM (Figure 4B) showed characteristic peaks at 3150 cm^−1^ (–OH and –NH_2_ stretching) and 1526 cm^−1^ (C=N). AA (Figure 4C) showed characteristic peaks at 3360 cm^−1^ (–COOH), 1700 cm^−1^, and 1650 cm^−1^ (C=O). Finally, the characteristic peaks of GM and AA superimposed the CSNP spectrum with a small shift (Figure 4D).

### 2.4. Antimicrobial Activity of Free and Loaded Gentamicin

The diameters of different inhibition zones indicated the qualitative antimicrobial activity. As shown in Table 1, in the first 24 h, GMCSNPs and GM–AA–CSNPs exhibited almost similar antibacterial activity to free GM. Subsequently, after 48 h, GMCSNPs and GM–AA–CSNPs showed much higher activity. On the other hand, CSNPs showed a significantly lower inhibition than free and nano-formulated drugs. 

**Figure 5:**
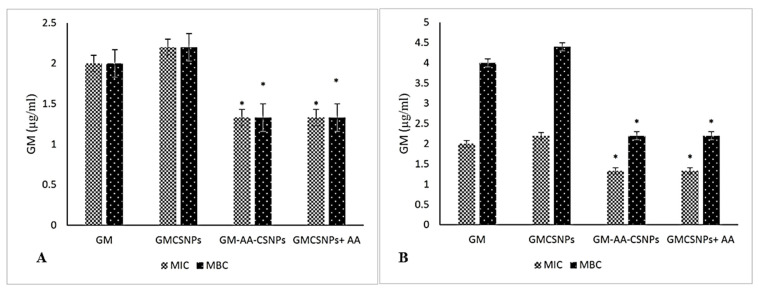
MICs and MBCs of free and loaded drugs for *S. aureus* (**A**) and *P. aeruginosa* (**B**). (*) represents the significant difference at *p* < 0.05 from the control and GM group.

The MIC and MBC results are illustrated in Figure 5. MIC and MBC values of the GM solution were 2 µg/mL for *S. aureus* and 2 µg/mL and 4 µg/mL for *P. aeruginosa*. The GMCSNPs exhibited almost the same activity, while GM–AA–CSNPs showed significantly enhanced MIC and MBC for both bacteria. In contrast, the free AA and CSNPs showed very high MICs of 200 and 500 µg/mL, respectively, and MBCs of 400 and 500 µg/mL (not shown in Figure 5). The physical mixture of free AA and GMCSNPs in the same equivalent concentration showed similar activity to GM–AA–CSNPs.

### 2.5. Cytotoxicity Assay

**Figure 6:**
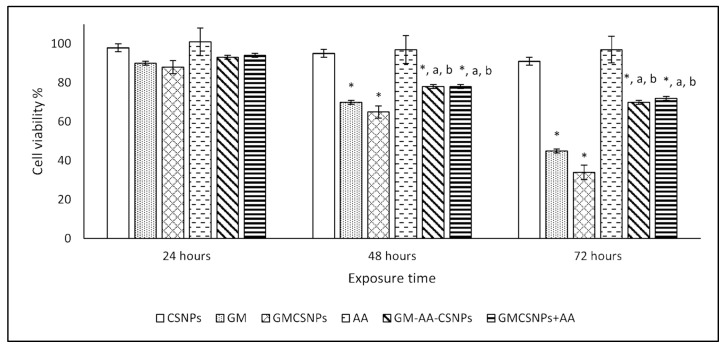
Cell viability % of HSF cells after exposure to CSNPs, GM, AA, GM–AA–CSNPs, and GMCSNPs/AA mixture at different times. (*, a, b) represents the significant difference at *p* < 0.05 from the control, GM, and GMCSNPs groups, respectively.

The cytotoxicity of the free and loaded drugs on the HSF human fibroblast normal cells was evaluated by MTT assay. The results are illustrated at different periods (24, 48, and 72 h) in Figure 6. After 24 h, the HSF cells did not show a considerable reduction in the viable cell number at the used concentration of all treatments compared with the control group. After 48 and 72 h, GM showed a significant decrease in cell viability to 70% and 45%, respectively. GMCSNPs exhibited a higher cytotoxic effect as they decreased the cell viability to 65% and 34% after 48 and 72 h, respectively. In contrast, after adding AA to GMCSNPs, the cell viability significantly increased to 78% and 70% after 48 and 72 h, respectively. GM–AA–CSNPs showed similar cell viability values. Finally, CSNPs and AACSNPs did not show cytotoxicity during the time of the experiment by the used concentration. 

### 2.6. Hemolysis Assay

**Figure 7:**
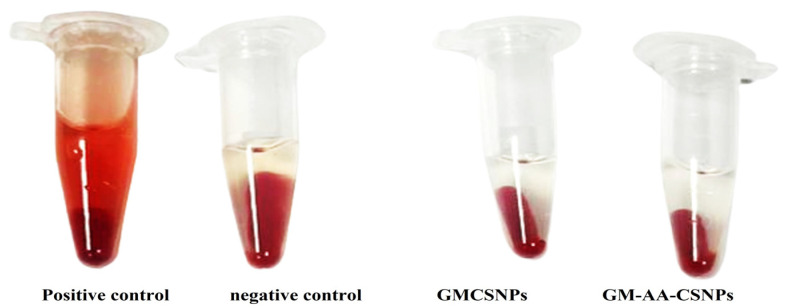
Hemolysis assay of GMCSNPs and GM–AA–CSNPs against the positive and negative controls.

The hemolytic properties of GMCSNPs and GM–AA–CSNPs were investigated to illustrate the in vitro biocompatibility. For both formulas, incubating blood with nanoparticles for an hour produced hemolytic ratios of 0.82% and 0.85, respectively (Figure 7).

## 3. Discussion

In the last decade, nano-drug delivery has been suggested as an exceptional approach to managing bacterial infection [16]. Nano-drug delivery allows a continuous release of antibiotics at the infection site. This unique behavior would help to reduce the required doses and decrease treatment-associated toxicity [17]. The formulation of GM-encapsulated nanoparticles has been investigated [7,8,14,18,19,20]. The results showed that using polymeric nanoparticles improved the antibacterial activity of gentamicin against the tested microorganisms. In the same framework, the current study aimed to prepare GM loaded in CSNPs to improve the antimicrobial activity of this broad-spectrum antibiotic.

Furthermore, the addition of AA may increase biocompatibility and minimize the side effects caused by GM administration. Ionic gelation is the most often used method in preparing CSNPs. Besides its simplicity, this method uses mild conditions and does not cause structural changes to the drug [21]. Thus, this method was used to perform our preparation. 

The potential toxicity and efficiency of nanoparticles for pharmaceutical applications mainly depend on their physicochemical properties [22]. The size and morphology of nanomaterials may affect their cellular uptake and distribution in the body [23], while the surface charge significantly determines the stability of nanoparticles and controls the interaction between biomolecules and nanoparticles [24]. 

In the current work, GM–AA–CSNPs were characterized regarding particle size, morphology, and zeta potential. The DLS measurements indicated an almost monodisperse size distribution. TEM examination showed that the nanoparticles have a spherical shape with a narrow distribution, revealing good agreement with the DLS findings and previously published results [25,26]. The zeta potential value was +30 mV, indicating our formula’s high stability in the aqueous medium. Additionally, the positive charge allows for the attachment of GM–AA–CSNPs to the negative bacterial surface [27]. Finally, the FTIR results of GM–AA–CSNPs showed that AA and GM were successfully loaded into the CSNPs.

The therapeutic effectiveness of the nano-formulated drug is related to the high EE% and LC% and a prolonged release profile [28,29]. Herein, Formula 3 (CS: drug ratio of 4:1:1) showed EE% values higher than 80% for both GM and AA in GM–AA–CSNPs, indicating the high efficiency of the carrier for loading both kinds of drugs. For a better comparison, the loading capacity (LC%) of different formulas was calculated. The results revealed that the highest LC% was also obtained at the CS: drug ratio of 4:1:1. Moreover, GM–AA–CSNPs showed prolonged release profiles over 60 h, allowing the release of 41%, 62%, or 80% of loaded drugs at pH 5, 6.4, and 7.5, respectively, which may provide a high blood concentration of GM and AA in vivo. Previous studies support our findings [15,30]. Interestingly, the infected wounds and bacterial infection-associated inflammation have acidic pH values [31]. Therefore, GM–AA–CSNPs are more suitable for treating infected wounds. However, the release of 41% GM at an alkaline pH may be acceptable in some conditions. 

*Staphylococcus aureus* is a Gram-positive pathogenic bacterium that causes aggressive infections such as soft tissue infections and pneumonia [32]. *Pseudomonas aeruginosa* is a Gram-negative opportunistic pathogenic bacterium that is one of the most common pathogens in urinary tract infections and wounds [33].

Gentamicin is an aminoglycoside antibiotic that exhibits bactericidal activity on Gram-negative bacteria and a few Gram-positive bacteria, such as *S. aureus* [34]. In bacterial cells, GM binds to the 16s rRNA at the 30s ribosomal subunit, disturbing mRNA translation to cause the formation of non-functional proteins [35]. In addition, GM causes the accumulation of reactive oxygen species due to the depletion of proteins involved with oxidation-reduction reactions, leading to bacterial death [36]. Ascorbic acid has antibacterial activity at 5–20 mg/mL concentrations [37]. Moreover, AA can modify the antimicrobial activity of various antibiotics and significantly decreases the adverse effects of ROS [38]. CSNPs exhibit antimicrobial activity due to the electrostatic interaction between the negatively charged cell surface (cell wall or outer membrane) of the bacteria and the positively charged amino group of chitosan [39]. 

Herein, in the first 24 h, using the same equivalent GM weight exhibited almost equal inhibition zones in the GM solution, GMCSNPs, and GM–AA–CSNPs, indicating the successful incorporation of the drug in the CSNP matrix without losing its biological activity. So, CSNPs can be usefully utilized in the delivery of GM. Moreover, GMCSNPs and GM–AA–CSNPs showed prolonged and enhanced antimicrobial activity, confirming the higher stability and sustainability of both nano-formulas. The present study is in close agreement with previous studies on the antibacterial activity of gentamicin-loaded polymeric nanoparticles against *P. aeruginosa* and *S. aureus* [8,40,41,42,43].

MIC and MBC are essential tests to evaluate the activity of antimicrobial drugs. Lower MIC and MBC values indicate higher activity of the drug used [44]. In the current study, the enhanced antimicrobial activity of GM–AA–CSNPs or the GMCSNPs/AA mixture revealed the synergistic effect of GM and AA. The synergistic effect of ascorbic acid with antibiotics is associated with protein synthesis inside bacterial cells, making the bacterial cells more permeable to antibiotics. In addition, the oxidation of AA releases hydrogen peroxide, which makes antibiotics more potent. Finally, AA may downregulate antibiotic-resistant genes. Our results were supported by the previous studies [45,46]

The choice of biodegradable and biocompatible materials for nano-drug delivery is a critical tactic for preventing their accumulation in the body and minimizing the probability of hazardous consequences [47]. Cell cultures are mostly utilized for screening purposes and for providing clear information about the toxicological profile of potential therapeutic drugs while avoiding the needless exposure of animals to extremely cytotoxic substances [48]. 

GM has been shown to trigger oxidative stress via the overproduction of ROS, which in turn damages macromolecules and subsequently causes cellular injury and necrosis [49]. Ascorbic acid is a potent natural antioxidant that can minimize oxidative stress, scavenge free radicals, and restabilize antioxidative systems. In many previous studies, AA has been used to reduce gentamicin-associated oxidative stress [10,45,50,51]

Herein, cytotoxic studies of free and loaded GM were performed on HSF human fibroblast cells. In agreement with the previous studies [28,29], the CSNPs showed no cytotoxic effect on HSF cells after 72 h of incubation. This finding confirmed the nontoxicity and biocompatibility of the used carrier. On the other hand, GM caused a significant cytotoxic effect, particularly in its nano-formula (GMCSNPs). The enhanced cytotoxicity of GMCSNPs may be due to the increased cellular uptake, leading to a higher ROS amount and more cellular damage [45]. 

Interestingly, GM–AA–CSNPs showed minimized cytotoxicity compared with GMCSNPs. Moreover, the addition of free AA induced a similar effect, confirming its protective effect. 

The hematological compatibility assessment of nanoparticles is critical for determining their toxicity and compatibility for in vivo applications [52]. Erythrocytes may act as osmometers, where changes in osmotic and physical conditions in the blood lead to their lysis. The released hemoglobin is easily measured by a colorimetric method [53]. In the current work, insignificant hemolysis was observed in GM–AA–CSNPs, indicating that the nanoparticles caused negligible damage to the erythrocyte membrane. These results indicated that the formulated nanoparticles might not adversely affect the blood cells and have acceptable blood compatibility.

## 4. Materials and Methods

### 4.1. Preparation of Chitosan-Loaded Gentamicin and Ascorbic Acid

Chitosan nanoparticles were formulated, as described in our previous work, by ionic gelation method using sodium tripolyphosphate (TPP) (0.1%, *v*/*v*) as a cross-linker [15]. In brief, a solution of chitosan (CS) of 200 mg in 100 mL acetic acid (2%, *v*/*v*) was prepared. After pH adjustment to 5.5, the CS solution was mixed with different volumes of the GM and AA solution (the CS ratios to drugs of 1:1, 2:1, and 4:1). The CSNPs formed after the dropwise addition of TPP under continuous stirring. The obtained nanoparticles were collected, lyophilized, and kept at −4 °C until use. The empty CSNPs were prepared by the same method without adding GM or AA.

### 4.2. Encapsulation Efficiency and Loading Capacity

The encapsulation efficiency (EE%) and loading capacity (LC%) were evaluated by vortexing 20 mg of the GM–AA–CSNPs with 5 mL 0.1 mol/L HCl for 30 min. The polymer residue was discarded, while the contents of GM and AA in the supernatant were determined by UV/VIS spectrophotometer (Shimadzu UV1800, Kyoto, Japan) at 248 nm and 297 nm, respectively. An empty CSNP sample was used as blank. The following equations calculated the EE% and LC%:

$$\mathrm{EE}\%=\;\frac{\mathrm{Loaded}\;\mathrm{amount}\;\mathrm{of}\;\mathrm{drug}}{\mathrm{The}\;\mathrm{initial}\;\mathrm{amount}\;\mathrm{of}\;\mathrm{drug}}\times 100$$

$$\mathrm{LC}\%=\frac{\mathrm{Loaded}\;\mathrm{amount}\;\mathrm{of}\;\mathrm{drug}}{\mathrm{The}\;\mathrm{dry}\;\mathrm{weight}\;\mathrm{of}\;\mathrm{CSNPs}}\times 100$$

The formula exhibiting the highest EE% and LC% was selected and used for further investigations.

### 4.3. In Vitro Release Studies

The in vitro drug release experiment was carried out as follows: suspensions of 30 mg GM–AA–CSNPs in 5 mL phosphate buffer solution (PBS) (pH 5, 6.5, and 7.5) were transferred in a dialysis bag (MWCO: 12,000). The dialysis bag was immersed in 50 mL PBS at 37 °C and shaken at 100 rpm. Triplicate independent samples were collected from each medium at a predetermined time and immediately replaced by PBS. The released GM and SA concentrations in PBS were colorimetrically measured by a UV/VIS spectrophotometer (Shimadzu UV1800). The cumulative release was calculated according to the following equation:

$$\mathrm{Cumulative}\;\mathrm{release}\;\mathrm{percentage}=\sum_{t=0}^{t}\frac{\mathrm{GM}(t)}{\mathrm{GM}(i)}\;\times \;100$$

where GM(i) and GM(t) are the initial concentration of GM and at specific times, respectively. 

### 4.4. Characterization of GM–AA–CSNPs

The physicochemical properties of GM–AA–CSNPs were fully described by Dynamic Light Scattering (DLS, Zeta sizer Nano ZS, Malvern Instruments, Worcestershire, UK), transmission electron microscopy (JEOL JAM-2100-HR-EM, Harwell, UK), and Fourier-transform infrared (FTIR) spectroscopy analyzer (FTIR-6100, JASCO, Tokyo, Japan). 

### 4.5. Antibacterial Activity Test

*Staphylococcus aureus* (ATCC 25923) and *Pseudomonas aeruginosa* (ATCC 27853) were obtained from the microbiology lab, College of Biotechnology, Misr University for Science and Technology, and were used to test the antimicrobial activity of the tested formula. The bacteria were inoculated into a nutrient broth medium at 37 °C on a rotary shaker at 200 rpm and incubated for 18 h. The bacterial stock cultures were maintained in their proper agar media at 4 °C and used for all microbiological tests. 

The antibacterial activity was evaluated by the well-diffusion method. Water solutions of GM and AA (100 µg/mL) and suspensions of CSNPs, GMCSNPs, AACSNPs, and GM–AA–CSNPs (100 µg/mL) in distilled water were prepared. Nine-milliliter Petri dishes containing sterilized media were separately inoculated with bacterial suspension (10^4^ CFU/mL). After media solidification, 1 mL diameter wells were punched by a sterile gel puncher and immediately filled with 100 µL of the tested materials. The plates were incubated at 37 °C for 24 h and 48 h. The antimicrobial activity was evaluated by determining the diameter of the inhibition zone around each well.

### 4.6. Minimum Inhibitory Concentration and Minimum Bactericidal Concentration

The minimum inhibitory concentration (MIC) experiment was performed using a broth microdilution test. Briefly, 100 µL of bacterial suspension (5000 CFU/mL) was mixed with 100 μL of serially diluted GM, AA, CSNPs, GMCSNPs, and GM–AA–CSNPs in addition to a mixture of GMCSNPs and AA. The concentrations of free and loaded drugs and empty carriers were equalized in all experiments. The plate was incubated for 24 h at 37 °C and a shaking speed of 120 rpm. The absorbances at 600 nm were measured using a microplate reader against a negative control. The minimum concentration that inhibits obvious bacterial growth is defined as MIC [54].

Afterward, a 10 µL aliquot of all tubes with no visible bacterial growth was used to inoculate Mueller Hinton agar plates. The plates were then incubated for 48 h at 37 °C. The minimum bactericidal concentration (MBC) was defined by the absence of growth on agar plates after specified incubation times.

### 4.7. Cytotoxicity

A normal human skin fibroblast cell line (HSF-1) was used to perform an MTT-cytotoxicity assay. The cells were maintained in complete DMEM media containing (10% FBS, a mixture of 100 U/mL penicillin, and 100 μg/mL streptomycin) and incubated at 37 °C under 5% CO_2_ and 95% humidity. Then, 100 µg/mL of the CSNPs, GM, AA, GMCSNPs, and GM–AA–CSNPs, in addition to a mixture of GMCSNPs and AA, were tested for their cytotoxicity. Typically, in 96-well plates, 1 × 10^5^ cells/well were exposed to the tested materials for 24 h. Then, the medium was removed, and the plates were rinsed using PBS (pH 7.2) before being inoculated with 100 µL MTT solution (0.5 mg/mL PBS) per well. The plates were incubated for four hours at 37 °C and received 100 µL DMSO per well. The cell viability was determined by measuring the absorbance at 570 nm using an ELISA reader apparatus (ELX-800n, Biotek, Winooski, VT, USA) and calculated according to the following equation:

$$\mathrm{Cell}\;\mathrm{viability}\;\%=\frac{\mathrm{The}\;\mathrm{absorbance}\;\mathrm{of}\;\mathrm{treated}\;\mathrm{cells}}{\mathrm{The}\;\mathrm{absorbance}\;\mathrm{of}\;\mathrm{untreated}\;\mathrm{cells}}\times 100$$

### 4.8. Biocompatibility Assay of GM–AA–CSNPs 

The biocompatibility assay was conducted as previously described [55]. Typically, in a test tube containing 3.2% sodium citrate, red blood cells (RBCs) were collected by centrifugation (1500 rpm, 10 min) of a human blood sample. The RBCs were washed three times with NaCl (0.9% *w*/*v*) and diluted to prepare a stock dispersion. Then, 50 µL of the stock dispersion was mixed with either 950 µL of GMCSNPs or GM–AA–CSNPs (100 µg/mL). The mixture was incubated at 37 °C for one hour before centrifugation at 10,000 rpm for five minutes. The hemolysis rate was assessed by measuring the absorbance of the supernatant at 540 nm against the negative control (saline solution) and positive control (distilled water). Finally, the hemolysis rate was calculated according to the following equation:

$$\mathrm{Hemolysis}\;\mathrm{rate}\;\%=\frac{\mathrm{Absorbance}\;\mathrm{of}\;\mathrm{sample}}{\mathrm{Absorbance}\;\mathrm{of}\;\mathrm{control}}\;\times 100$$

### 4.9. Statistical Analysis

All experiments were based on three independent measurements. Results are presented as mean ± standard deviation. Statistical comparisons were carried out by analysis of variance using Graph Pad Prism 7 for the Windows-10 computer software package and Dunnett’s multiple comparisons test at α = 0.05. A *p*-value of ≤0.05 was considered statistically significant.

## 5. Conclusions

The current study was conducted in the framework of sustainable development goals. A novel formula containing gentamicin and ascorbic acid was successfully prepared and tested for its antimicrobial activity, cytotoxicity, and biocompatibility. The prepared formula successfully released 40% to 82% of GM, depending on the medium pH. Compared to free GM, both GMCSNPs and GM–AA–CSNPs exhibited enhanced antimicrobial activity after 48 h of exposure to bacteria in vitro. On the other hand, GM–AA–CSNPs showed lower MIC and MBC values against *S. aureus* and *P. aeruginosa* than free GM and GMCSNPs. Moreover, the cell viability assay confirmed ascorbic acid’s effect in minimizing GM cytotoxicity. Finally, our formula showed excellent biocompatibility. Therefore, it can be favorable for the delivery of gentamicin. However, more in vivo studies are essential in the future.

## Figures and Tables

**Table 1:** Diameters of inhibition zones of free and loaded drugs against *Staphylococcus aureus* and *Pseudomonas aeruginosa*.

Drug (100 µg/mL)	*Staphylococcus aureus* Inhibition Zone (mm) after (24/48 h)	*Pseudomonas aeruginosa* Inhibition Zone (mm) after (24/48 h)
CSNPs	8/8	9.5/9.7
GM	24.0/24.2	21.5/21.5
GMCSNPs	23.4/32.1	20.1/29.4
GM–AA–CSNPs	24.2/34.0	21.8/31.7

## Data Availability

The data presented in this study are available on request.
